# A Bright Organic Fluorophore for Accurate Measurement of the Relative Quantum Yield in the NIR‐II Window

**DOI:** 10.1002/smll.202411866

**Published:** 2025-02-24

**Authors:** Hanchen Shen, Xinyan Zhu, Jianyu Zhang, Changhuo Xu, Jacky W. Y. Lam, Ben Zhong Tang

**Affiliations:** ^1^ Department of Chemistry Hong Kong Branch of Chinese National Engineering Research Center for Tissue Restoration and Reconstruction Department of Chemical and Biological Engineering Division of Life Science, and State Key Laboratory of Molecular Neuroscience The Hong Kong University of Science and Technology Clear Water Bay, Kowloon Hong Kong 999077 China; ^2^ MOE Frontiers Science Center for Precision Oncology Faculty of Health Sciences University of Macau Macao 999078 China; ^3^ School of Science and Engineering Shenzhen Institute of Aggregate Science and Technology The Chinese University of Hong Kong Shenzhen (CUHK‐Shenzhen) Shenzhen Guangdong 518172 China

**Keywords:** organic fluorescent dye, photoluminescence quantum yield, photoluminescence standard, second near‐infrared emission, twisted intramolecular charge transfer

## Abstract

Organic dyes with photoluminescence in the second near‐infrared window (NIR‐II, 1000–1700 nm) are promising for bioimaging and optoelectronic devices. Photoluminescence quantum yield (PLQY) is a direct measure of their performance. Integrating sphere technology is effective in determining the absolute PLQY. However, the low PLQY values of most NIR‐II organic fluorophores lead to significant measurement errors. Therefore, the most common method for PLQY determination is a relative approach using a photoluminescence spectrometer and a standard reference like IR‐26. Although the relative method enables precise calculation of the PLQY ratio between the sample and the reference, the specific PLQY value of IR‐26 is not clearly defined and is reported to range from 0.05% to 0.50%. Such a deviation can cause significant errors in relative PLQY measurements. In this study, it is reported that a bright organic fluorophore called TPE‐BBT exhibits a high PLQY of 3.94% in THF, which can be accurately measured using a commercially available integrating sphere. Using TPE‐BBT as a standard, the PLQY values of IR‐26 in 1,2‐dichloroethane and IR‐1061 in dichloromethane are accurately determined to be 0.0284% and 0.182%, respectively. It is hoped that using this reliable standard will unify the evaluation criteria for NIR‐II organic fluorophores.

## Graphical abstract

The THF solution of TPE‐BBT exhibits bright emission in the NIR‐II window with a PLQY of 3.94%. As a PLQY standard, TPE‐BBT features a well‐defined chemical structure, superior stability, and ease of use. TPE‐BBT is successfully used as a reference to determine the PLQY of IR‐26 (0.0284% in 1,2‐dichloroethane) and IR‐1061 (0.182% in dichloromethane).

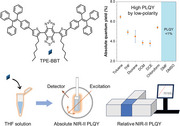

## 1 Introduction

In vivo fluorescence bioimaging can provide real‐time insights into complex physiological and pathological processes, making it invaluable for both biological research and modern medicine.^[^ ^1^ ^]^ However, its practical applications are often hindered by the complex light‐matter interactions within biological environments, which can result in tissue autofluorescence and photon scattering, ultimately reducing the signal‐to‐background ratio and imaging quality. Fortunately, researchers have discovered that photons within the second near‐infrared (NIR‐II, 1000–1700 nm) window exhibit minimal interactions with biological tissues. This property enables high‐resolution imaging with deep tissue penetration and a low‐noise background.^[^ ^2^, ^3^ ^]^ Nowadays, NIR‐II materials have been widely used in advanced applications like fluorescent probes,^[^ ^4^ ^]^ sensors for medical diagnostics,^[^ ^5^, ^6^ ^]^ and phototheranostics.^[^ ^7^, ^8^ ^]^ Besides, NIR‐II materials have also found applications in areas such as organic light‐emitting diodes,^[^ ^9^, ^10^ ^]^ communication technologies,^[^ ^11^ ^]^ and information security displays.^[^ ^12^, ^13^ ^]^ Although they exhibit limitations in stability, color purity, and short emission lifetime,^[^ ^14^ ^]^ organic NIR‐II fluorophores offer several advantages over their inorganic counterparts, including higher biosafety, more tunable optical properties, and greater environmental friendliness.^[^ ^15^ ^]^ More importantly, their well‐defined chemical structures ensure satisfactory reproducibility for basic research on NIR‐II applications. One key parameter that determines the performance of organic fluorophores is the photoluminescence quantum yield (PLQY), which is defined as the ratio of the number of emitted photons to the number of absorbed photons:^[^ ^16^ ^]^

(1) $$\begin{array}{c} \Phi =\frac{\mathrm{numb}\mathrm{er}\;\mathrm{of}\;\mathrm{phot}\mathrm{ons}\;e\mathrm{mitt}\mathrm{ed}}{\mathrm{numb}\mathrm{er}\;\mathrm{of}\;\mathrm{phot}\mathrm{ons}\;a\mathrm{bsor}\mathrm{bed}}\; \end{array}$$

This quantity directly evaluates the photoluminescence (PL) efficiency and guides the selection of organic emitters. Compared to fluorophores emitting visible light, NIR‐II fluorophores typically have a much narrower energy gap between the HOMO (Highest Occupied Molecular Orbital) and LUMO (Lowest Unoccupied Molecular Orbital). According to the energy gap law, a narrower energy gap typically results in stronger vibronic coupling between the ground and excited states, leading to a lower PLQY.^[^ ^17^ ^]^ As optimizing optoelectronic performance is of central importance for developing new NIR‐II materials, the determination of PLQY is becoming a necessity in the photophysical and mechanism study of every NIR‐II organic fluorophore.

**Figure 1:**
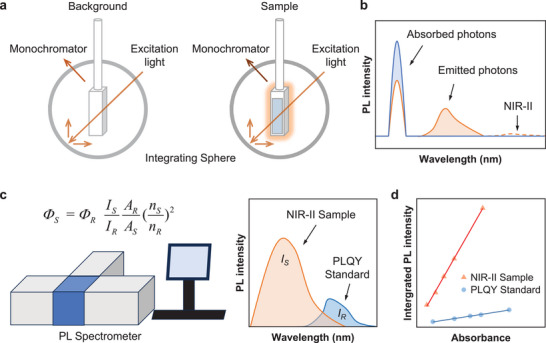
a) Schematic illustration of an integrating sphere setup and its working mechanism for the absolute PLQY measurement. b) Spectral scans by the monochromator. The blue graph refers to the incident light, and the orange graph refers to the sample. The blue and orange areas represent the absorbed and emitted photons, respectively. The orange dashed line demonstrates that the low PLQY of the NIR‐II sample can lead to significant testing errors. c) Schematic illustration of the relative PLQY measurement. d) Schematic illustration of the relative PLQY calculation using a linear regression of the sample and reference, respectively.

Ideally, the PLQY value can be determined by directly measuring the emitted and absorbed photons. This method yields what researchers commonly refer to as absolute PLQY. The absolute method directly reflects the exact PLQY and thus receives increasing attention. Since the work of Vavilov was published in 1924, researchers have been searching for an accurate method to measure the absolute PLQY.^[^ ^18^ ^]^ After ≈100 years of development, integrating sphere spectroscopy has emerged as the most reliable and mature technique (**Figure** **1**a).^[^ ^19^ ^]^ This technology measures changes in both incident light (blue color, absorbed photons) and photoluminescence (orange color, emitted photons) before and after sample loading (Figure 1b). The PLQY can then be directly calculated from these measurements. Recently, integrating sphere setups have become commercially available. Especially, integrating spheres from Hamamatsu Photonics can detect photons in a wavelength range from 350 to 1650 nm, greatly facilitating the measurement of PLQY of NIR‐II materials. Unfortunately, most NIR‐II organic fluorophores display a very low PLQY, far below 1% (dashed line in Figure 1b), resulting in an extremely low signal‐to‐noise ratio (SNR). This leads to difficulties in precisely measuring the number of emitted photons with an integrating sphere and a significant error in the PLQY calculation. In this work, we define “precision” as the good repeatability of a test with sufficiently low random errors, ensuring reliable measurements. “Accuracy,” in contrast, refers to how close the measured value is to the actual value, which can be affected by systematic errors in the test environment and instrument setting. While integrating sphere setup is a proven commercial technology with good accuracy, poor precision in NIR‐II emissive materials measurements significantly reduces measurement reliability. A large number of replicates may help bring the average test value closer to the actual value. However, it is difficult to ensure sufficient replication numbers each time in practical testing, and the commonly used number of replicates (such as 3–5 times) cannot guarantee a slight deviation between measured and actual values, leading to inaccurate results. Since a higher SNR improves precision, we believe that testing samples with higher PLQYs will produce more accurate NIR‐II absolute PLQY results.

An alternative method is the relative method, which measures the PL spectra of a given sample and a specific reference with a known absolute PLQY (Figure 1c). In our practical operations, compared to the integrating sphere, the PL spectrometer is often more sensitive to emitted photons, which ensures a much better measurement of SNR and precision. Taking TPA‐BBT as an example,^[^ ^20^ ^]^ some molecules with relatively high PLQY can produce strong NIR‐II signals when using the PL spectrometer but fail to show any observable signals when using the integrating sphere (Figure S1, Supporting Information). Thus, the relative method can measure PLQY more precisely even if it is less than 1%. The fundamental principle of the relative quantum yield method states that when two solutions absorb an equal number of photons, the ratio of their integrated fluorescence intensities under identical measurement conditions (excitation wavelength, excitation bandwidth, emission bandwidth, and integration time) corresponds to the ratio of their PLQYs (Figure 1c). Then, the relative PLQY of the sample can be calculated using Equation (2), where the subscripts *S* and *R* represent the sample and reference, respectively. *Φ_R_* is the known PLQY of the reference standard, *I* is the integrated PL intensity, *A* is the absorbance of the solution at the excitation wavelength, and *n* is the refractive index of the solvent used. Notably, when the absorbance value is small, the ratio of absorbed photon numbers in the equation can be approximated by the ratio of the absorbance values. Therefore, Equation (2) can be rewritten as the commonly used formula Equation (3). In principle, only a single solution of the sample and reference should be enough. However, to reduce the error, multiple solutions of the sample and reference at different concentrations are commonly required. Then Equation (3) can be rearranged into a linear equation Equation (4). By plotting *I_S_* versus *A_S_* and *I_R_* versus *A_R_*, the ratio of the “gradients” can be used to calculate the PLQY of the sample (Figure 1d). Based on Equations ((2), (3), (4)), we can see that the relative PLQY measurement can determine *Φ_S_*/*Φ_R_*, which is the ratio of the PLQY of the sample to that of the reference. Ultimately, an accurate PLQY value of the reference is required to determine the PLQY of the sample accurately.

(2) $$\begin{array}{c} \;\Phi_{S}=\Phi_{R}\;\frac{I_{S}}{I_{R}}\;\left(\frac{1-10^{-A_{R}}}{1-10^{-A_{S}}}\right){\left(\frac{n_{S}}{n_{R}}\right)}^{2}\; \end{array}$$

(3) $$\begin{array}{c} \;\Phi_{S}=\Phi_{R}\;\frac{I_{S}}{I_{R}}\;\frac{A_{R}}{A_{S}}\;{\left(\frac{n_{S}}{n_{R}}\right)}^{2}\; \end{array}$$

(4) $$\begin{array}{c} \;\Phi_{S}=\Phi_{R}\;\frac{Grad_{S}}{Grad_{R}}\;{\left(\frac{n_{S}}{n_{R}}\right)}^{2}\; \end{array}$$

However, commercially available PLQY standards in the NIR‐II window display very low PLQY values. Taking a widely used NIR‐II PLQY standard called IR‐26 as an example, its PLQY has been reported to range from 0.05% to 0.50%.^[^ ^21^ ^]^ Accurate determination of such a low PLQY value is challenging using an absolute method. When using this standard, the measured PLQY values of NIR‐II materials can deviate from the actual values by 10 times.^[^ ^20^, ^22^, ^23^, ^24^, ^25^, ^26^, ^27^ ^]^ On the other hand, researchers adopt various reference standards, which may not reliably correlate with each other.^[^ ^28^, ^29^, ^30^, ^31^ ^]^ This could make the PLQY values in different reports in the literature completely incomparable. As a result, relative PLQY measurements can only be described as “precise” rather than “accurate.” Therefore, developing new NIR‐II PLQY standards with reliable PLQY values is an urgent task. In addition, certified NIR‐II PLQY standards are valuable tools for validating the performance of integrating sphere setups and the reliability of other ensembles and methods used for measuring PLQY.

In this work, we developed a new PLQY standard for NIR‐II materials using a NIR‐II aggregation‐induced emission (AIE) luminogen called TPE‐BBT.^[^ ^32^ ^]^ The transparent solutions using low‐polarity organic solvents exhibited high PLQY values, and the toluene solution of TPE‐BBT demonstrated the highest PLQY (exceeding 6%) when measured with an integrating sphere. To the best of our knowledge, this is among the highest absolute PLQY values reported for NIR‐II emissive organic small‐molecule dye solutions. Then, we prepared the THF solution of TPE‐BBT with a moderate PLQY of ≈4% as the standard for the following experiments. We demonstrated that, at room temperature (20 °C), no aggregation was observed when the 808 nm absorbance ranged from 0.01 to 0.1, and the concentration‐dependent PLQY variations were within the normal instrument error range. Using the TPE‐BBT solution as a standard, whose PLQY was determined to be 3.94 ± 0.25%, we successfully calibrated the PLQY for two commercially available NIR‐II PLQY standards, namely IR‐1061 and IR‐26.

## 2 Results and Discussion

**Figure 2:**
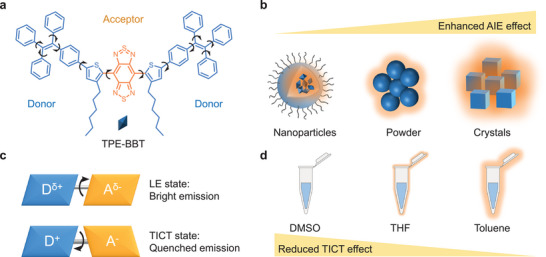
a) Chemical structure of TPE‐BBT. b) Schematic illustration of the AIE effect of TPE‐BBT. c) Schematic illustration of the TICT process. d) Schematic illustration of the enhanced PL intensity of TPE‐BBT solution as the solvent polarity decreases.

TPE‐BBT is an AIE luminogen (AIEgen) showing ultrahigh PLQY.^[^ ^32^, ^33^ ^]^ The synthetic route and structural characterization are provided in the Supporting Information. Its structure consists of a benzobisthiadiazole (BBT) electron acceptor and an electron donor comprising a bulky thiophene *π* bridge and a tetraphenylethylene (TPE) moiety (**Figure** **2**a). TPE‐BBT exhibits a significant AIE effect. The molecule exhibits only weak fluorescence when dissolved in the good solvent DMSO. However, upon formation of nanoparticles, the emission intensifies significantly due to the restriction of molecular motion, resulting in an absolute PLQY of ≈2%. As the aggregation degree further increases, via forming amorphous powders or even crystals, the PLQY continues to rise, exceeding 10% in the crystalline state (Figure 2b).

The high PLQY values suggest that TPE‐BBT could be a reliable PLQY standard for NIR‐II materials. In addition, TPE‐BBT exhibits absorption from 600 to 900 nm and fluorescence from 850 to 1500 nm, aligning well with the typical features of NIR‐II materials. However, ensuring consistency and reproducibility is challenging during the preparation of TPE‐BBT nanoparticles, and different research laboratories may not achieve a fixed PLQY. Comparatively, simply dissolving TPE‐BBT in its good solvent to prepare a solution sample as a PLQY standard would be better. As a NIR‐II emissive organic fluorophore, TPE‐BBT has a typical donor–acceptor (D–A) electronic structure. Therefore, its emission is greatly influenced by environmental polarity. When the polarity is high, the molecule will adopt a twisted conformation to stabilize the charge separation in the excited state, which is the emission‐quenching twisted intramolecular charge transfer (TICT) effect (Figure 2c).^[^ ^34^ ^]^ Theoretically, if TPE‐BBT is provided with a low‐polarity environment, charge separation can be suppressed, promoting better electron overlap in the locally excited (LE) state to achieve enhanced emission (Figure 2d). Therefore, we selected a series of good solvents with different polarities for TPE‐BBT to study its solvent effect.

**Figure 3:**
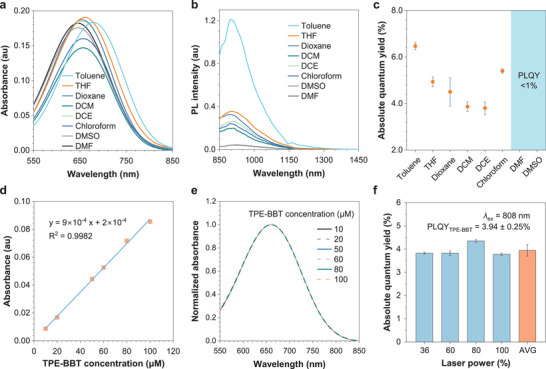
a) Absorption spectra of TPE‐BBT in various organic solvents. [TPE‐BBT] = 10 µm. b) PL spectra of TPE‐BBT in various organic solvents. [TPE‐BBT] = 10 µm. THF: tetrahydrofuran, DCM: dichloromethane, DCE: 1,2‐dichloroethane, DMF: dimethylformamide, DMSO: dimethyl sulfoxide. c) Absolute PLQYs of various TPE‐BBT solutions measured by an integrating sphere setup. [TPE‐BBT] = 10 µm. Data represent mean ± S.D.; *n* = 3. d) Plots of the absorbance at 808 nm versus the TPE‐BBT concentration with the linear regression relationship. e) Normalized absorption spectra of TPE‐BBT in THF with different concentrations. f) Absolute PLQYs of the THF solutions of 100 µm TPE‐BBT in the 850–1500 nm range under various laser power. *λ*_ex_ = 808 nm. Data represent mean ± S.D.; *n* = 4.

We first measured the absorption and emission spectra of TPE‐BBT in different solvents. Given the critical importance of cuvette type and measurement geometry in the emission spectra measurement, we have included a photograph of the cuvette and a simplified diagram of the PL measurement geometry in Figure S2 (Supporting Information). As shown in **Figure** **3**a,b, TPE‐BBT shows the most redshifted absorption and the strongest PL intensity in the low‐polarity toluene. In solvents of medium polarity (tetrahydrofuran (THF), dioxane, dichloromethane (DCM), 1,2‐dichloroethane (DCE), and chloroform), the absorption of TPE‐BBT is slightly blueshifted, accompanied by a decrease in fluorescence. The use of highly polar solvents like dimethyl sulfoxide (DMSO) and dimethylformamide (DMF) leads to further blueshifts in the absorption peaks, and these solvents cause a significant decrease and a slight redshift in fluorescence. These results indicate that TPE‐BBT exhibits an obvious TICT effect. We then measured the PLQYs of TPE‐BBT in different solvents using an 808 nm laser excitation, which is commonly used for NIR‐II bioimaging (Figure 3c).^[^ ^4^ ^]^ In the low‐polarity toluene, TPE‐BBT has the highest PLQY (> 6%). In solvents of medium polarity, TPE‐BBT also shows high PLQYs of ≈4–5%. In highly polar solvents such as DMSO, we could not measure a reliable PLQY, indicating that the integrating sphere fails to detect enough NIR‐II photons at this point.

Based on these results, we can select organic solvents with low to medium polarity to prepare TPE‐BBT solutions as new PLQY standards for NIR‐II materials. Given that the bright luminescence of the toluene solution could potentially impact the accuracy of the relative PLQY measurement, we opted to utilize the THF solution of TPE‐BBT as the standard for this study. Generally, for relative PLQY testing, it is necessary to keep the absorbance at the excitation wavelength between 0.01 and 0.1. Figure 3d shows that the molecular concentration range corresponding to the absorbance range is ≈10–100 µm. After normalizing the absorption spectra at different concentrations, we found no noticeable changes, indicating no dye aggregation or new species formation (Figure 3e). Subsequently, we examined the PLQY of the TPE‐BBT solution within this concentration (Figures S3a and S4–S8, Supporting Information). All measurement results fall within the range of 3–5%, with large error bars observed for the 10 and 25 µm samples. Repeated tests showed that the data for the 10 µm sample exhibited noticeable fluctuations (Figure S9, Supporting Information), indicating a large instrument error for low‐concentration samples (Figure S3b, Supporting Information). To further verify the data stability of the 100 µm sample data, we tested its PLQY under varying laser powers and observed small fluctuations (Figures 3f; Figure S10, Supporting Information). We hypothesize that at higher sample concentrations, the increased PL intensity reduces instrumental errors. We also examined the effects of photothermal conversion on the PLQY measurement. The results showed that under normal testing conditions, where the operating time was ≈90 s, the temperature increase for the 100 µm sample irradiated with 100% laser power was less than 5 °C (Figure S11, Supporting Information). This represents the maximum photothermal effect achievable when testing the TPE‐BBT solution using the optical setup of the Hamamatsu integrating sphere. Notably, to eliminate the changes in photothermal effects caused by differences in laser irradiation area, we ensured that the laser spot was entirely positioned on the sample during the photothermal experiments. As a result, the product of the power density (W cm^−2^) and the irradiation area (cm^2^) equals the laser power (W), which is 1.5 W when the laser machine is set to 100%. To further eliminate potential temperature‐related influences, we also examined the PLQY values across a more extensive temperature range. Directly measuring the temperature‐dependent absolute PLQY change in the TPE‐BBT solution presents a challenge for our integrating sphere setup. To address this, we employed a relative method. UV–vis absorption spectra of TPE‐BBT in THF reveal that the absorbance at 808 nm remains constant between 20 and 60 °C (Figure S12a, Supporting Information). Based on this observation, we measured the temperature‐dependent PL spectra of TPE‐BBT in THF to investigate the effect of temperature on PLQY. As shown in Figure S12b,c (Supporting Information), the PL spectra show minimal change across various temperatures, and the integrated PL intensities exhibit no significant variation. These results suggest that temperature changes have a negligible impact on the PLQY measurements. The weak photothermal effect and insensitivity to the temperature change explain why changing laser power does not affect the PLQY measurements (Figure 3f). Additionally, there was no significant change in PLQY for solutions prepared using THF bubbled with air or nitrogen (Figures S3c and S13, Supporting Information), indicating a minimal influence of the oxygen content. These findings indicate that the PLQY data for 100 µm TPE‐BBT are reliable. The average PLQY value obtained for 100 µm TPE‐BBT in THF under different laser powers is 3.94%, which is even higher than that of its nanoparticle form. Ultimately, the PLQY of 3.94% was established as the standard for the THF solution of TPE‐BBT. The excitation wavelength was then switched to 730 nm to further confirm the PLQY value (Figure S14, Supporting Information). The results indicate that the data acquired is also ≈4%, which further validates the reliability of this PLQY value (Figure S15a, Supporting Information). It is noteworthy that TPE‐BBT exhibits excellent shelf stability. We prepared the organic solution of the solid powder of TPE‐BBT that had been on the shelf for two years and measured the absorption spectrum. It was found that the spectrum was nearly identical to that of freshly prepared TPE‐BBT (Figure S15b, Supporting Information). All these results prove that the THF solution of TPE‐BBT can act as a PLQY standard for the accurate measurement of relative PLQY in the NIR‐II window.

**Figure 4:**
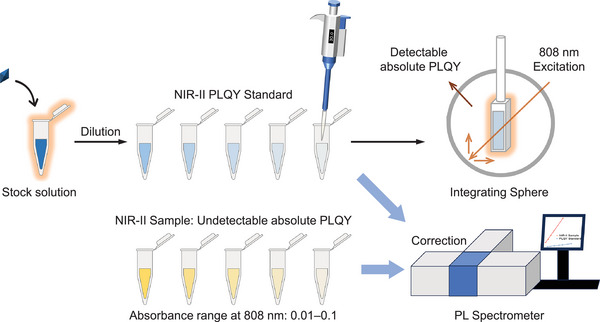
Schematic illustration of the procedure of the relative PLQY measurement using the THF solution of TPE‐BBT as the reference.

The TPE‐BBT solution was then used as the reference for the relative PLQY measurement. The operation procedure is shown in **Figure** **4**. We first prepared a TPE‐BBT stock solution of ≈1 mm using THF and then further diluted it with THF to achieve a series of concentrations from 10 to 100 µm. Subsequently, UV–vis spectroscopy is used to confirm that the absorbance of these TPE‐BBT solutions at 808 nm was within the range of 0.01–0.1. These absorbance values should be accurately recorded. Then, a relative PLQY measurement can be conveniently conducted using a PL spectrometer and the calculation equation to accurately evaluate the samples with low PLQY or when no integrating sphere setup is available. Following a similar process, a series of solutions of the test samples are prepared with absorbance at 808 nm within the range of 0.01–0.1. It is important to note that due to the existing discrepancies in molar absorptivity, the molecular concentrations of the sample and reference may differ significantly. The concentration of the NIR‐II samples must be strictly determined based on absorbance. Next, a PL spectrometer will be used to measure the spectra of the TPE‐BBT and NIR‐II sample solutions under identical conditions. As the emission wavelengths of the samples and references may differ, the emission correction should be applied to the PL spectrophotometer. After plotting the spectral integral area against the absorbance for each compound, linear regression analysis was performed to calculate the gradient. The gradient values, along with the refractive index factors of the solvents for both the sample and the reference, were then applied to Equation (4) to calculate the relative PLQY of the NIR‐II sample.

**Figure 5:**
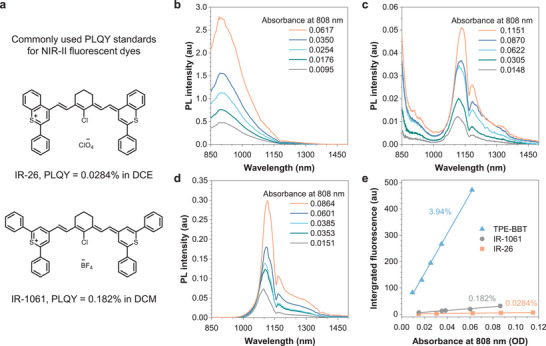
a) Chemical structures of IR‐26 and IR‐1061. (b–d) PL spectra of b) TPE‐BBT in THF, c) IR‐26 in DCE, and d) IR‐1061 in DCM with different absorbance values at 808 nm. e) Plots of the integrated PL spectra of TPE‐BBT (850–1500 nm in THF), IR‐1061 (850–1500 nm in DCM), and IR‐26 (850–1500 nm in DCE) against the absorbance.

As a demonstration, we used this procedure to measure the relative PLQYs of two NIR‐II dyes, namely IR‐26 and IR‐1061 (**Figure** **5**a). These two dyes are commonly used for relative PLQY measurement for 808 nm excitable NIR‐II materials. Many researchers have attempted to measure their absolute PLQYs, but the low PLQY values have made it challenging to obtain accurate results.^[^ ^35^, ^36^, ^37^ ^]^ Therefore, this causes a significant deviation of the relative PLQY from the actual PLQY value, thus making the measurement lose its physical significance. Herein, we use TPE‐BBT as a reference to measure the relative PLQYs of IR‐26 and IR‐1061. On the one hand, we can verify the reliability of TPE‐BBT as a new PLQY standard. On the other hand, we can have access to the accurate absolute PLQYs of IR‐26 and IR‐1061, giving the relative PLQY calculated from them practical physical significance and comparability. The comparable PLQYs of NIR‐II materials will greatly promote the development and communication of the field of NIR‐II materials. Figure 5b–d demonstrate the PL spectra of TPE‐BBT (THF solution), IR‐26 (DCE solution), and IR‐1061 (DCM solution), respectively. Notably, while the PL spectra of TPE‐BBT, IR‐1061, and IR‐26 vary significantly in wavelength, adjusting the *y*‐axis of the PL spectra for TPE‐BBT demonstrates that it emits sufficient photons within the PL wavelength range of IR‐26 and IR‐1061 (Figure S16, Supporting Information). Furthermore, the corrected integrated PL intensity is directly proportional to the emitted photon numbers. Therefore, TPE‐BBT can serve as a reliable standard for various NIR‐II emissive materials. Additionally, The baseline lifting in the 850–1000 nm range for IR‐26 is due to normal background noise amplified by the correction. Since IR‐26 does not exhibit fluorescence in the 850–1000 nm range,^[^ ^22^, ^35^, ^36^ ^]^ we actually calculated the integrated fluorescence intensity in the 1000–1500 nm range of IR‐26 to avoid the impact of the correction. After calculating the gradients of the linear regression lines of the integrated fluorescence intensity on the absorbance, we can determine the PLQY of IR‐26 to be 0.0284% and IR‐1061 to be 0.182% by substituting the PLQY value of TPE‐BBT as 3.94% (Figure 5e). We think that this result is reliable because when we substitute the new PLQY value of IR‐26 into the relative PLQY calculation, the significant discrepancy between the absolute and relative PLQY in our previous work is greatly reduced.^[^ ^32^ ^]^ In that work, using IR‐26 with a PLQY of 0.5% as a reference, the absolute PLQY of TPE‐BBT nanoparticles is calculated to be 1.8%, while the relative PLQY is 31.5%. In comparison, when substituting the PLQY value of IR‐26 as 0.0284%, the relative PLQY of TPE‐BBT nanoparticles is 1.8%, matching well with their absolute PLQY.

## 3 Conclusion

In conclusion, we have measured the absolute PLQY of TPE‐BBT in THF to be 3.94% using an integrating sphere instrument. The high PLQY and suitable absorption and emission wavelength suggest that TPE‐BBT could be a reliable PLQY standard for NIR‐II materials. The TPE‐BBT solution exhibits negligible concentration‐dependent PLQY changes within the absorbance range of 0.01–0.1 at 808 nm at room temperature, and no aggregation was observed within this range. Using the TPE‐BBT solution as a standard, we successfully measured and calibrated the PLQY for two commercially available NIR‐II PLQY standards, namely IR‐26 (PLQY = 0.0284% in DCE) and IR‐1061 (PLQY = 0.182% in DCM). The calibrated value of IR‐26 enables the relative PLQY measurements to show only slight deviations from the absolute value, accurately reflecting the PLQY of the samples. Our results confirm that the THF solution of TPE‐BBT can serve as a reliable PLQY standard for NIR‐II fluorophores.

## Conflict of Interest

The authors declare no conflict of interest.

## Supporting information



Supporting Information

## Data Availability

The data that support the findings of this study are available from the corresponding author upon reasonable request.
