# Supplementary material for: A Bright Organic Fluorophore for Accurate Measurement of the Relative Quantum Yield in the NIR‐II Window
Source: Small. 2025 Feb 24;21(13):2411866. doi: 10.1002/smll.202411866 (PMC11962700; doi:10.1002/smll.202411866)
Supplement: Supplementary file 1 — Supporting Information [file SMLL-21-2411866-s001.pdf]

NANO · MICRO
small

Supporting Information

for *Small*, DOI 10.1002/smll.202411866

A Bright Organic Fluorophore for Accurate Measurement of the Relative Quantum Yield in the NIR-II Window

Hanchen Shen, Xinyan Zhu, Jianyu Zhang, Changhuo Xu, Jacky W. Y. Lam* and Ben Zhong Tang**

Supporting Information

A Bright Organic Fluorophore for Accurate Measurement of the Relative Quantum Yield in the NIR-II Window

Hanchen Shen, Xinyan Zhu, Jianyu Zhang, Changhuo Xu, Jacky W. Y. Lam,* and Ben Zhong Tang**

Experimental sections

Materials and instruments

TPE-BBT was synthesized by the procedure reported in our previous work (Reference [32] in the main text). IR-26 was purchased from Luxottica / Exciton. IR-1061 was purchased from Sigma-Aldrich. Anhydrous toluene, anhydrous tetrahydrofuran (THF), anhydrous dioxane, anhydrous dichloromethane (DCM), anhydrous 1,2-dichloroethane (DCE), anhydrous dimethylformamide (DMF), and anhydrous dimethyl sulfoxide (DMSO) were purchased from Energy-Chemical. HPLC grade chloroform was purchased from Scharlau. UV-vis-NIR absorption spectra were measured using a Shimadzu UV-Visible Spectrophotometer UV-2600i. PL spectra were recorded on a Horiba Fluorolog-3 (MODEL: FL3C-111) Spectrofluorometer equipped with a NIR-II detector (MODEL: DSS-IGA020L). Absolute fluorescence quantum yields (QY) were measured on a Hamamatsu Quantum Yield Spectrometer C13534 Quantaurus-QY Plus.

Absolute PLQY measurement

Accurately weigh 1.2 mg of TPE-BBT solids and dissolve them completely in 1 mL of THF to obtain a stock solution of TPE-BBT with a concentration of 1 mM. In subsequent tests, the stock solution can be diluted to the required concentration with THF. The Hamamatsu integrating sphere (Quantaurus-QY Plus) was then used to determine the absolute PLQYs of TPE-BBT solutions. The excitation source was provided by 808-nm or 730-nm laser setups, and the excitation and emission spectra were measured using BT-CCD and InGaAs linear image sensors. Based on this assembled setup, the integrating sphere can detect photons from 350 to

1650 nm. The PLQY for each sample in the 850–1500 nm range was measured three or four times using a reference cuvette.

Relative PLQY measurement

1 mg of TPE-BBT solids were completely dissolved in 1 mL of THF. The resulting solution was then diluted using THF to achieve an absorbance between 0.06 and 0.1 at 808 nm. Subsequently, samples were prepared by diluting the TPE-BBT solution with THF in volume ratios of approximately 5:1, 3:1, 1:1, 1:3, and 0:1. The prepared samples should have absorbance values from 0.01 to 0.1 at 808 nm. Next, five TPE-BBT solutions were transferred into 10-mm path fluorescence cuvettes, and their emission spectra were recorded using a Horiba Fluorolog-3 Spectrofluorometer with an 808-nm laser excitation. Notably, the PL spectra correction and blank subtraction are required to remove the wavelength-dependent detection efficiency from measured spectra. The emission spectra of five samples were integrated from 850 to 1500 nm. Similar procedures were performed for IR-26 and IR-1061, respectively. The integrated fluorescence intensities of TPE-BBT in THF, IR-1061 in DCM, and IR-26 in DCE were plotted against absorbance at 808 nm and the plots were fitted into their corresponding linear functions. The gradients of these lines were employed in the calculation of their PLQY values based on the following equation:

$$\Phi_S = \Phi_R \frac{Grad_S}{Grad_R} \left(\frac{n_S}{n_R}\right)^2$$

The sample refers to IR-1061 or IR-26; the reference is TPE-BBT; n_S and n_R are the refractive indices of the used solvent for the sample and reference, respectively.

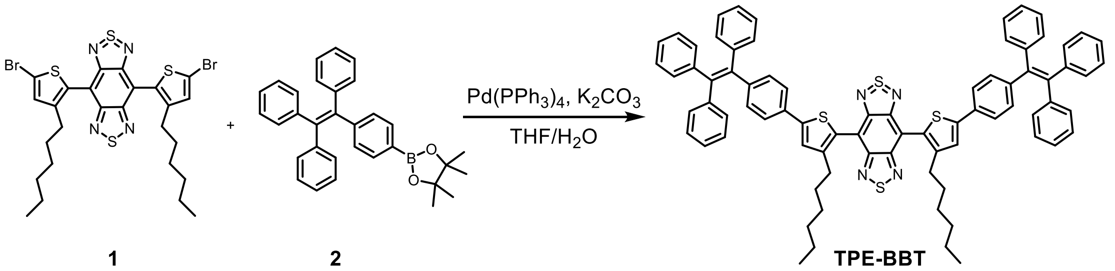

1 2 TPE-BBT

Synthetic routes to TPE-BBT. TPE-BBT was synthesized by Suzuki Coupling. Typically, **1** (500 mg, 0.73 mmol), **2** (837 mg, 1.83 mmol), $Pd(PPh_3)_4$ (84 mg, 0.073 mmol) and K_2CO_3 (353 mg, 2.56 mmol) in the THF/water mixture (V/V = 5/1, 60 mL) were heated at 70 °C under nitrogen atmosphere for 6 h. After cooling to room temperature, the mixture was extracted with DCM for 3 times and dried over anhydrous Na_2SO_4. The filtrate was concentrated under reduced pressure and was further purified by silica gel column chromatography with DCM and

hexane to afford the desired product as deep-blue solid (399 mg, 46%). ^{1}H NMR (400 MHz, Chloroform-*d*) δ (ppm) 7.45 (d, J = 8.4 Hz, 4H), 7.40 (s, 2H), 7.18–7.03 (m, 34H), 2.59–2.51 (m, 4H), 1.59 (q, J = 7.6 Hz, 4H), 1.65–1.56 (m, 12H), 0.73 (t, J = 6.9 Hz, 6H).

Statistical Analysis

The pre-processing of data and sample size (n) for each statistical analysis were clearly noted in the figure captions. The quantitative data were expressed as mean ± standard deviation (S.D.). The statistical comparisons were made using analysis of variance (one-way ANOVA) by GraphPad Prism 8. P value < 0.05 was considered statistically significant, while P > 0.05 was considered not significant (ns). Figures and linear regression analysis were made by OriginPro 2023.

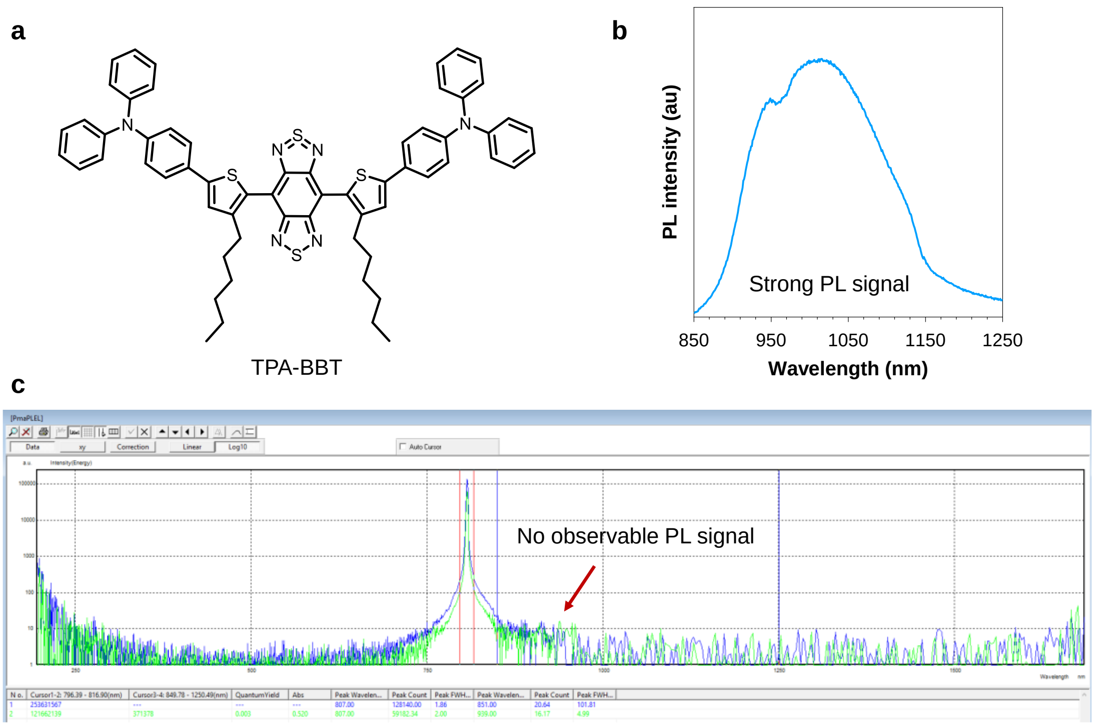

Figure S1. (a) Chemical structure of TPA-BBT. (b) Photoluminescence (PL) spectrum of TPA-BBT in the THF/water mixture with the water fraction of 99%. [TPA-BBT] = 10 μM; λ_{ex} = 808 nm. (c) Absolute PLQY measurement of TPA-BBT in THF/water mixtures with the water fraction of 99%. [TPA-BBT] = 10 μM; λ_{ex} = 808 nm.

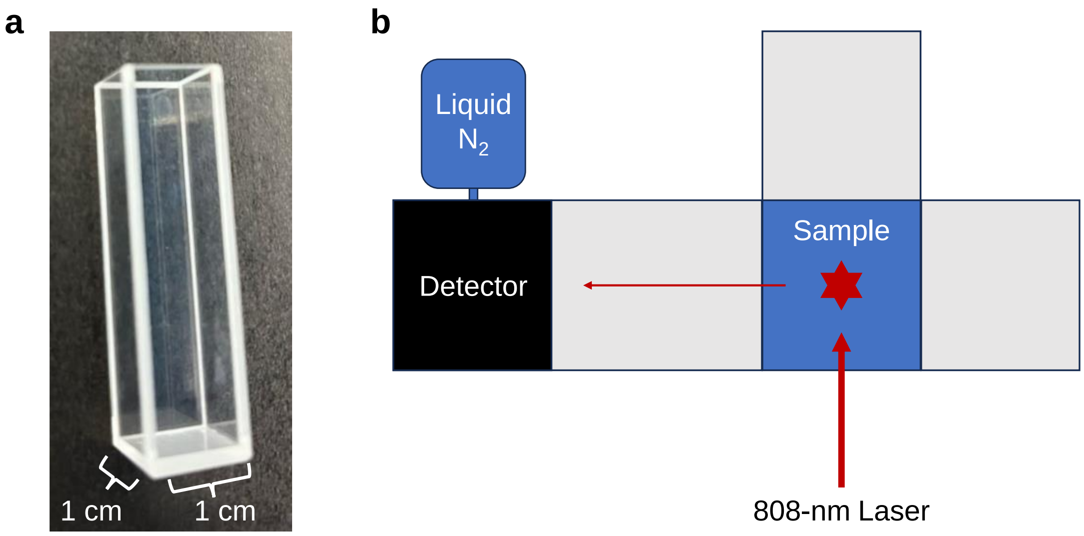

Figure S2. (a) Photograph of the cuvette used for PL spectra measurements. (b) Schematic illustration of the PL measurement geometry.

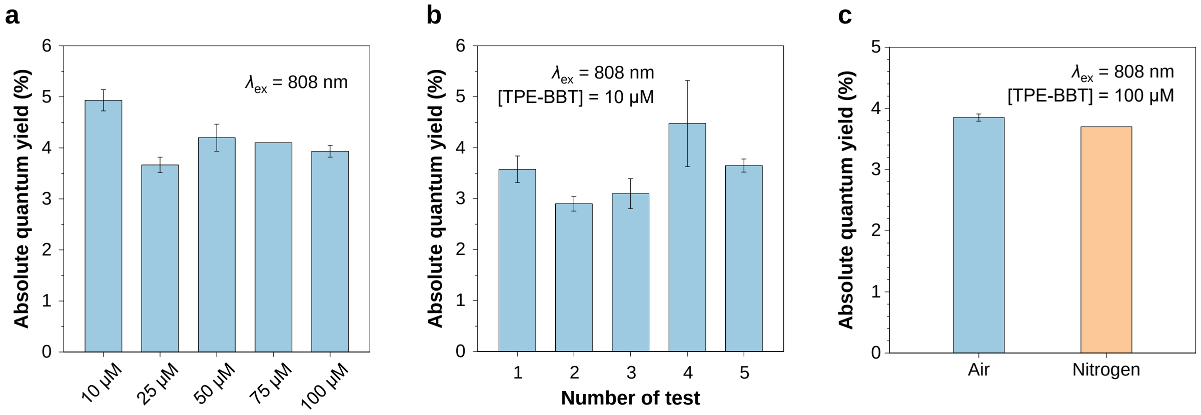

Figure S3. (a) Absolute PLQYs of the THF solutions of TPE-BBT with various concentrations in the range of 850–1500 nm. λ_{ex} = 808 nm. Data represent mean ± S.D.; n = 3. (b) Absolute PLQYs of the THF solutions of 10 μM TPE-BBT in the range of 850–1500 nm for multiple times. λ_{ex} = 808 nm. Data represent mean ± S.D.; n = 4. (c) Absolute PLQYs of the THF solutions of 100 μM TPE-BBT in the range of 850–1500 nm bubbled with air or N_2. λ_{ex} = 808 nm. Data represent mean ± S.D.; n = 4.

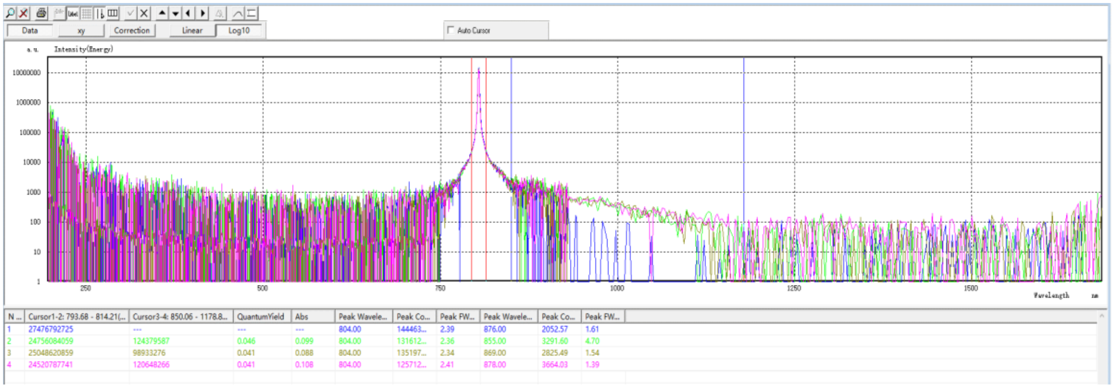

Figure S4. Absolute PLQY measurement of 10 μM of TPE-BBT in THF. λ_{ex} = 808 nm; n = 3.

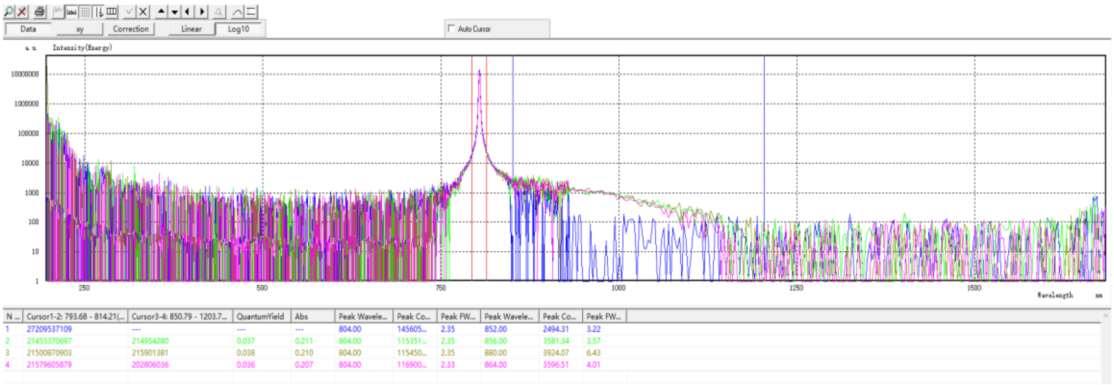

Figure S5. Absolute PLQY measurement of 25 μM of TPE-BBT in THF. λ_{ex} = 808 nm; n = 3.

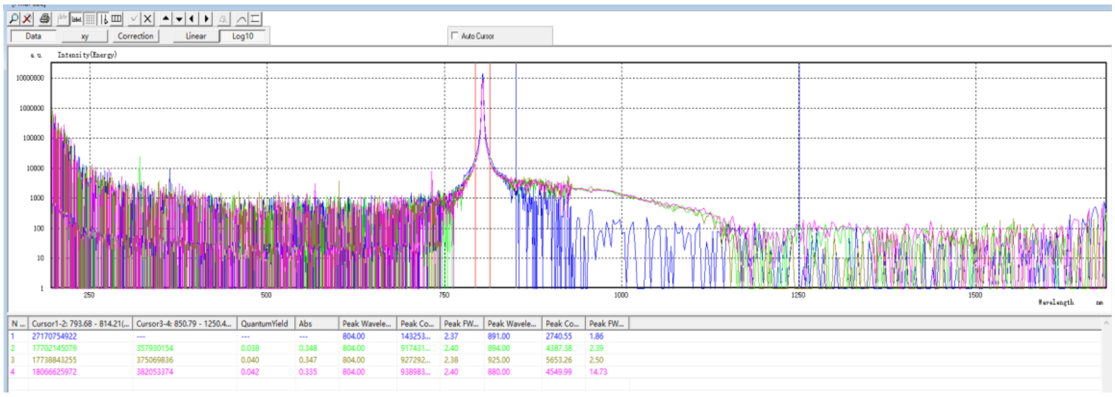

Figure S6. Absolute PLQY measurement of 50 μM of TPE-BBT in THF. λ_{ex} = 808 nm; n = 3.

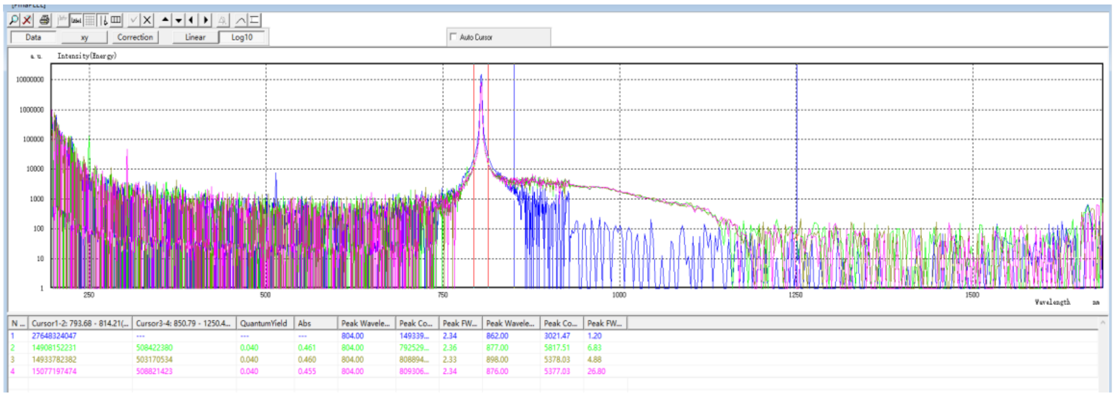

Figure S7. Absolute PLQY measurement of 75 μM of TPE-BBT in THF. λ_{ex} = 808 nm; n = 3.

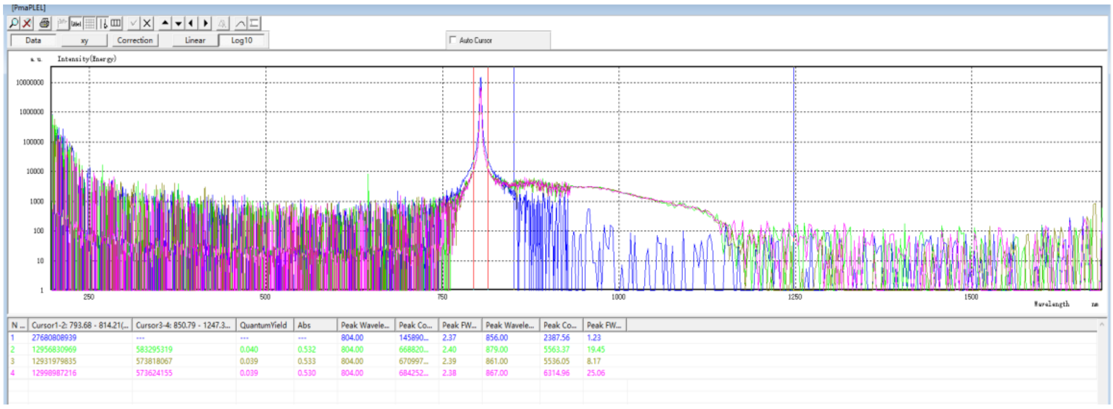

Figure S8. Absolute PLQY measurement of 100 μM of TPE-BBT in THF. λ_{ex} = 808 nm; n = 3.

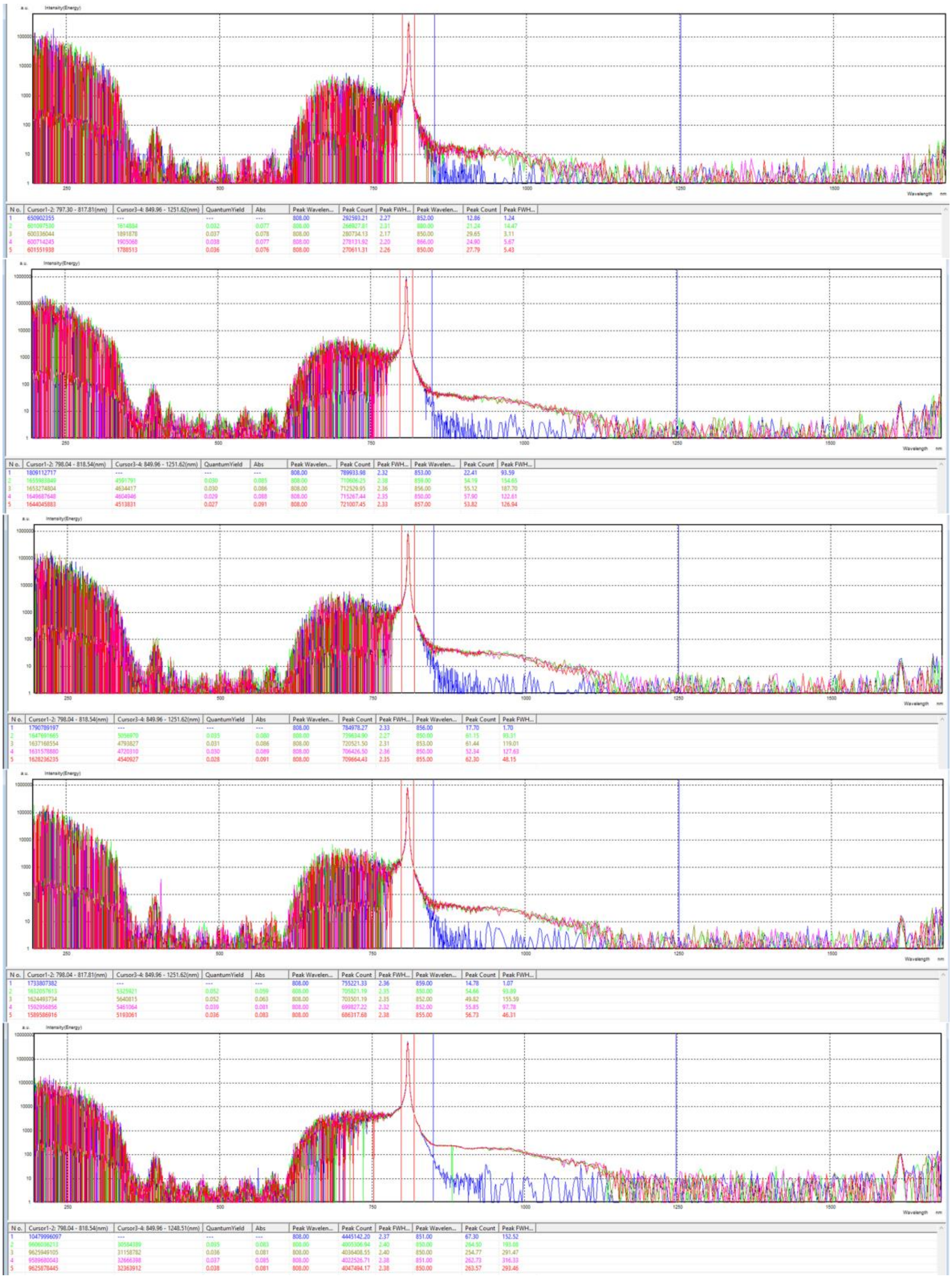

Figure S9. Absolute PLQY measurement of 10 μM of TPE-BBT in THF for five times. λ_{ex} = 808 nm; n = 4.

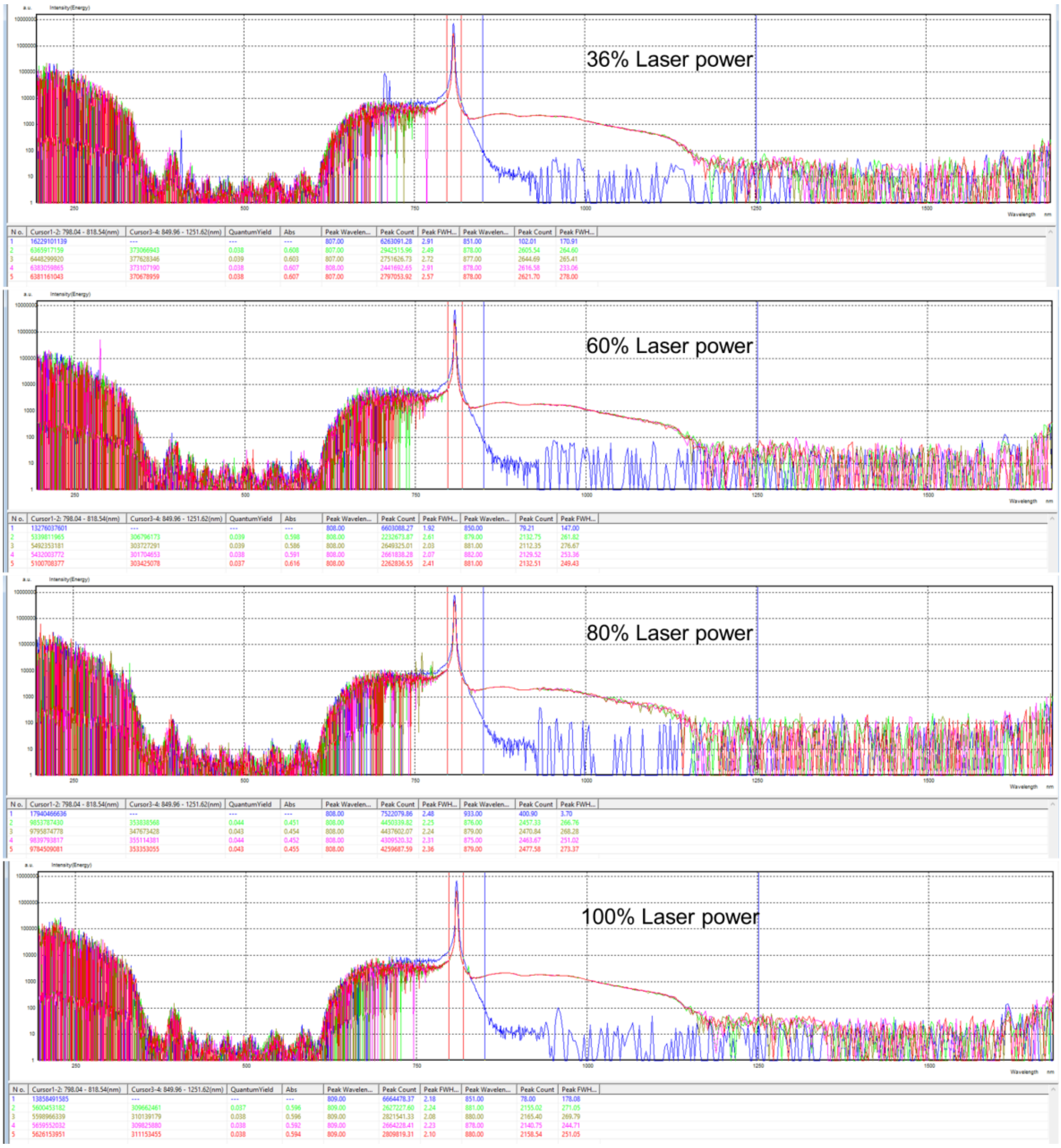

Figure S10. Absolute PLQY measurement of 100 μM of TPE-BBT in THF using different laser powers. λ_{ex} = 808 nm; n = 4.

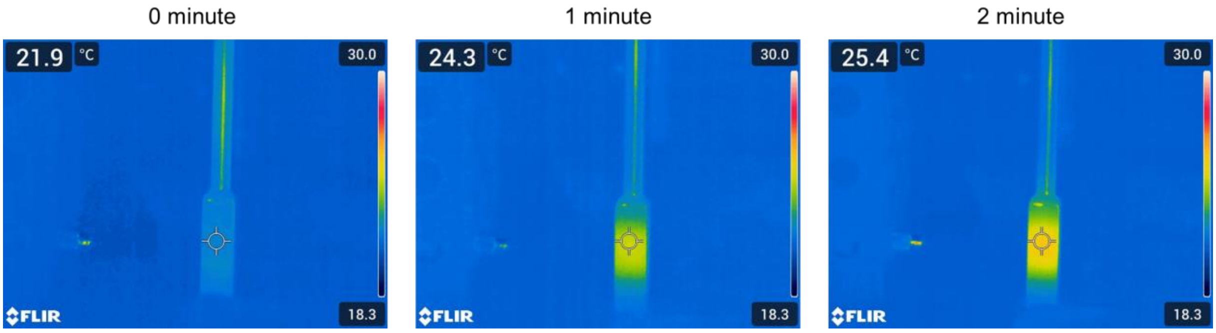

Figure S11. Infrared thermal images of 100 μM TPE-BBT in THF before and after NIR-laser irradiation (808 nm, 1.5 W). The entire laser spot was directed onto the sample, guaranteeing that all the light power was delivered to the sample.

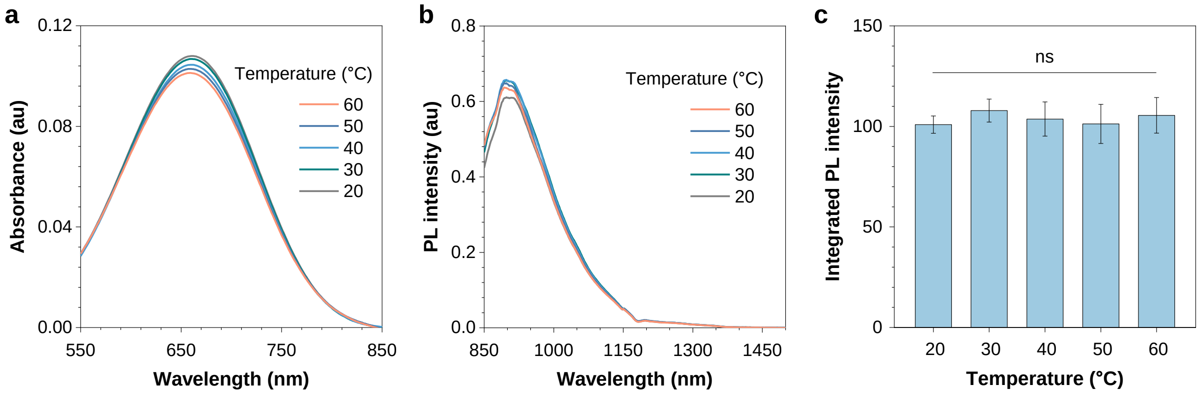

Figure S12. (a) UV-Vis absorption and (b) PL spectra of TPE-BBT in THF at different temperatures. (c) Integrated PL intensities of TPE-BBT (850−1500 nm in THF) solutions at different temperatures. [TPE-BBT] = 10 μM. λ_{ex} = 808 nm. Data represent mean ± S.D.; n = 3. $P > 0.05$ is indicated by "ns" for not significant using the one-way ANOVA with a Tukey multiple comparisons test.

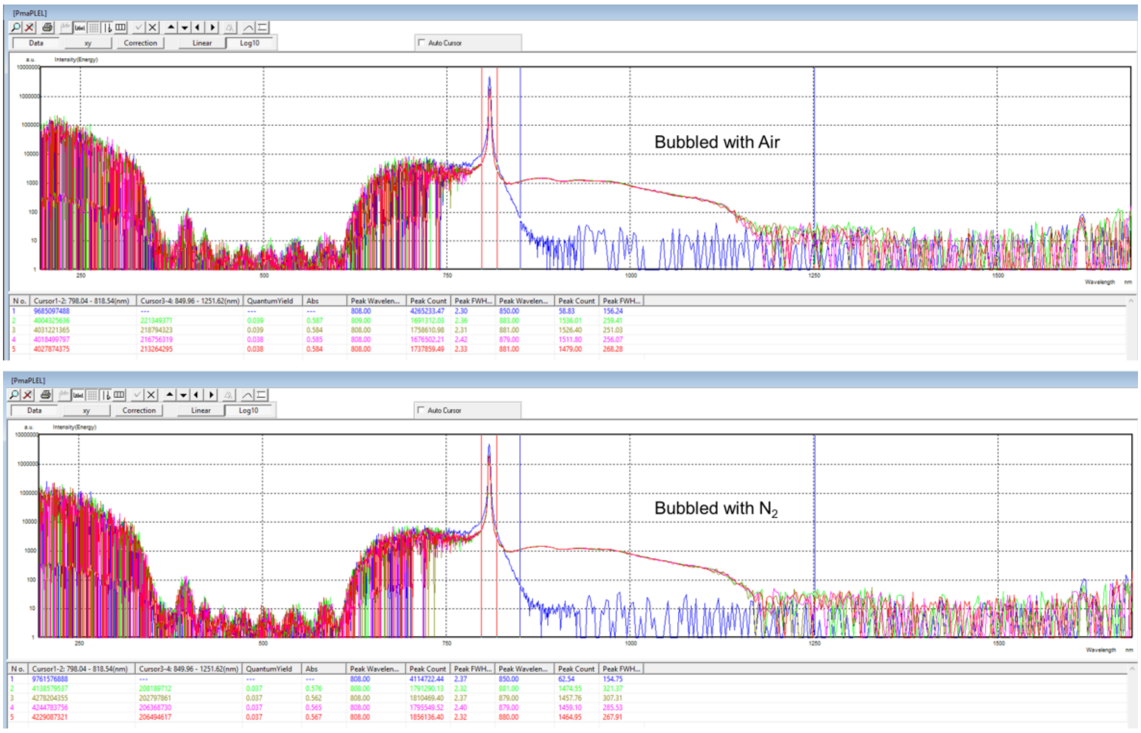

Figure S13. Absolute PLQY measurement of 100 μM of TPE-BBT in THF bubbled with air or N_2, respectively. λ_{ex} = 808 nm; n = 4.

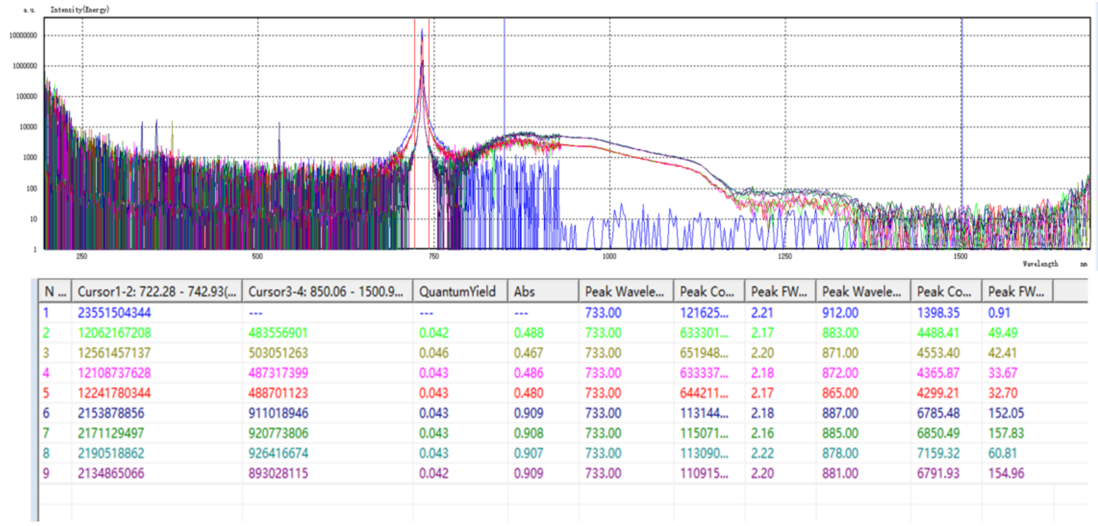

N ...	Cursor1-2: 722.28 - 742.93(...	Cursor3-4: 850.06 - 1500.9...	QuantumYield	Abs	Peak Wavele...	Peak Co...	Peak FW...	Peak Wavele...	Peak Co...	Peak FW...
1	23551504344	---	---	---	733.00	121625...	2.21	912.00	1398.35	0.91
2	12062167208	483556901	0.042	0.488	733.00	633301...	2.17	883.00	4488.41	49.49
3	12561457137	503051263	0.046	0.467	733.00	651948...	2.20	871.00	4553.40	42.41
4	12108737628	487317399	0.043	0.486	733.00	633337...	2.18	872.00	4365.87	33.67
5	12241780344	488701123	0.043	0.480	733.00	644211...	2.17	865.00	4299.21	32.70
6	2153878856	911018946	0.043	0.909	733.00	113144...	2.18	887.00	6785.48	152.05
7	2171129497	920773806	0.043	0.908	733.00	115071...	2.16	885.00	6850.49	157.83
8	2190518862	926416674	0.043	0.907	733.00	113090...	2.22	878.00	7159.32	60.81
9	2134865066	893028115	0.042	0.909	733.00	110915...	2.20	881.00	6791.93	154.96

Figure S14. Absolute PLQY measurement of 10 μM (No. 2 to 5) or 100 μM (No. 6 to 9) of TPE-BBT in THF. λ_{ex} = 730 nm; n = 4.

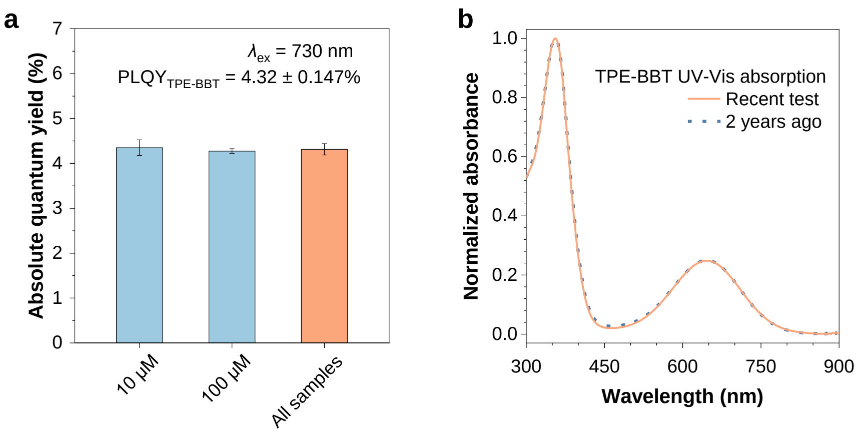

Figure S15. (a) absolute quantum yields of TPE-BBT solutions with different concentrations in the range of 850–1500 nm. λ_{ex} = 730 nm. Data represent mean ± S.D.; n = 3. (b) Normalized absorption spectra of the THF solution of TPE-BBT prepared by freshly prepared TPE-BBT and TPE-BBT that has been stored on the shelf for two years.

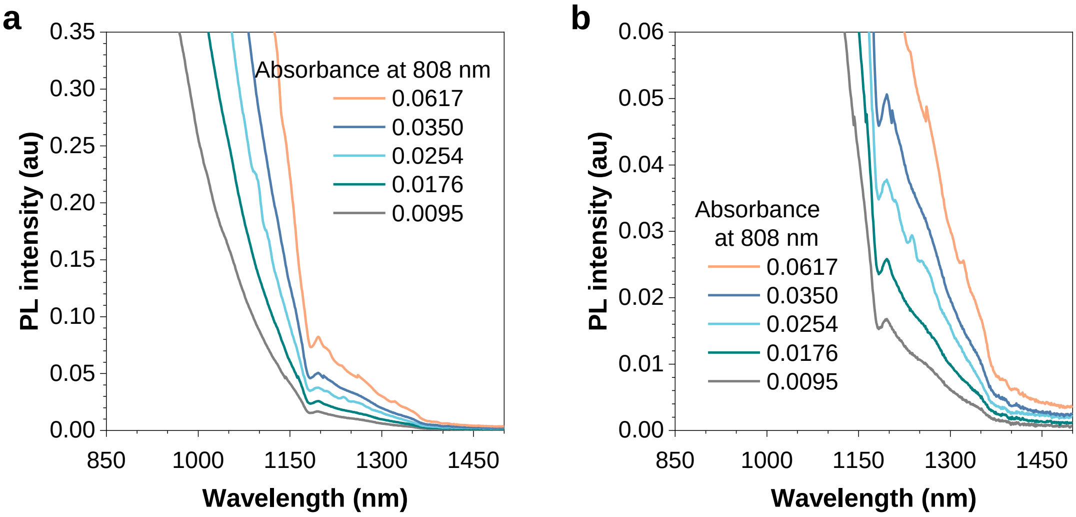

Figure S16. PL spectra of TPE-BBT in THF with the y-axis scaled to match the PL intensity ranges of (a) IR-1061 and (b) IR-26, as shown in Figures 5c and 5d.
